# Identification of multiple TAR DNA binding protein retropseudogene lineages during the evolution of primates

**DOI:** 10.1038/s41598-022-07908-8

**Published:** 2022-03-09

**Authors:** Juan C. Opazo, Kattina Zavala, Luis Vargas-Chacoff, Francisco J. Morera, Gonzalo A. Mardones

**Affiliations:** 1 Integrative Biology Group, Universidad Austral de Chile, Valdivia, Chile; 2 Instituto de Ciencias Ambientales y Evolutivas, Facultad de Ciencias, Universidad Austral de Chile, Valdivia, Chile; 3 Millennium Nucleus of Ion Channel-Associated Diseases (MiNICAD), Valdivia, Chile; 4 Instituto de Ciencias Marinas y Limnológicas, Universidad Austral de Chile, Valdivia, Chile; 5 Centro Fondap de Investigación de Altas Latitudes (IDEAL), Universidad Austral de Chile, Valdivia, Chile; 6 Applied Biochemistry Laboratory, Facultad de Ciencias Veterinarias, Instituto de Farmacología y Morfofisiología, Universidad Austral de Chile, Valdivia, Chile; 7 Department of Physiology, School of Medicine, Universidad Austral de Chile, Valdivia, Chile; 8 Center for Interdisciplinary Studies of the Nervous System (CISNe), Universidad Austral de Chile, Valdivia, Chile

**Keywords:** Evolutionary genetics, Molecular evolution

## Abstract

The TAR DNA Binding Protein (TARDBP) gene has become relevant after the discovery of its several pathogenic mutations. The lack of evolutionary history is in contrast to the amount of studies found in the literature. This study investigated the evolutionary dynamics associated with the retrotransposition of the TARDBP gene in primates. We identified novel retropseudogenes that likely originated in the ancestors of anthropoids, catarrhines, and lemuriformes, i.e. the strepsirrhine clade that inhabit Madagascar. We also found species-specific retropseudogenes in the Philippine tarsier, Bolivian squirrel monkey, capuchin monkey and vervet. The identification of a retropseudocopy of the TARDBP gene overlapping a lncRNA that is potentially expressed opens a new avenue to investigate TARDBP gene regulation, especially in the context of TARDBP associated pathologies.

## Introduction

The availability of whole-genome sequences has accelerated research on the evolution of different genetic elements. Together with genomic DNA-based gene duplication, an important source of evolutionary innovation are the events of RNA retrotranscription and its insertion into the genome^1,2^. In mammals, retrotranscription depends on the long interspersed nuclear element 1 (L1 or LINE1) enzymatic machinery encoded by retrotransposable elements, which generate an intronless gene duplicate that could produce a protein similar to the parental counterpart^3^. However, most retrotranscribed sequences are inserted at a random position in the genome, lacking all necessary transcription elements and becoming a pseudogene, a phenomenon called “dead on arrival”^3^. However, because a significant number of retrocopies are located in introns of other genes, they have potential to regulate their host genes functioning as antisense transcripts^4–6^. On the other hand, in the human genome, a number of retrocopies overlap with long noncoding RNAs (lncRNAs)^7^, which are regulatory noncoding RNAs of > 200 nucleotides^8^. lncRNAs can establish specific interactions with nucleic acids and proteins, acting in diverse fashions as critical regulators of gene expression in several biological processes, including pathological conditions such as cancer and neurodegenerative disorders^9^.

Retrocopies are abundant in placental mammals, especially in primates^10^. Extensive evidence indicates that their presence is related to several types of diseases, including neurodegenerative disorders^11^. Because retrocopies have potential to produce harmful effects on genomes and transcriptomes, silencing mechanisms seem to have evolved to restrict retrotransposition. Intriguingly, during the life of healthy humans the brain is the only known somatic tissue where retrotransposition is de-repressed^12^. Therefore, identifying the presence of retrocopies/retropseudogenes is not only an important piece of information to have a complete picture of the evolution of any particular gene, but also is necessary to fully understand human health.

The TAR DNA Binding Protein (TARDBP) gene, which encodes the Transactive response DNA-binding protein 43 kDa (TDP-43), has gained considerable attention after the initial discovery that its mutations can cause familial amyotrophic lateral sclerosis (ALS) and frontotemporal dementia (FTD), two major forms of neurodegenerative disorders^13,14^, with ALS being the most frequent motor neuron disorder in adults^15^. Up to date, more than 50 pathogenic missense mutations have been characterized^13,14^. TDP-43 is an RNA-binding protein with a variety of RNA metabolism functions, including transcription, mRNA transport and stabilization, miRNA biogenesis, lncRNA processing, and translation^16^. More recent findings indicate that TDP-43 participates in the pathogenesis of other neurodegenerative disorders of several other proteinopathies, such as Parkinson’s disease and Alzheimer’s disease, which are conditions characterized by toxic protein aggregation^17^. In human cells, under physiological conditions, TDP-43 mainly localizes in the nucleus, but in neurons and glial cells of ALS and FTD patients it shuttles and accumulates in the cytoplasm where eventually aggregates and contribute to the onset and progression of these diseases^18–22^.

The TARDBP gene is conserved in species that share a common ancestor deep in time^23^, suggesting that this gene carries out essential functions. This gene underwent an event of positive selection in the ancestor of mammals^24^, suggesting functional adaptations for the group. More recently in evolutionary time, it has been shown that during the evolution of humans, genes related to diseases like Alzheimer's also underwent positive selection^25^. Although events of positive selection are seen as conferring selective advantage, as a by-product, they can also have adverse effects^26^. In this regard, it is proposed that human susceptibility to neurodegenerative disorders could be a consequence of improving our cognitive function^27,28^. Besides these studies, not much is known regarding the evolution of TARDBP in primates. Understanding the evolutionary history of genes represents a critical piece of information, among other things, to perform meaningful comparisons and to understand the variation in function in different species. However, evolutionary studies have been primarily directed to the functional copy, while much less is known of other phenomena that could potentially impact the functions associated with the gene. In this regard, the evolution of primates is characterized by a peak of retrotransposition activity in the anthropoid ancestor^29,30^, which left a signature of intronless copies, functional or not, of a number of genes in the genome.

The aim of this study is to investigate the retrotransposition dynamics associated with the TARDBP gene in primates. According to our phylogenetic and synteny analyses, we identified retropseudogenes that originated at different times during the evolution of primates. TARDBP retropseudogenes originated in the anthropoid ancestor, between 67 and 43.2 million years ago, in the ancestor of catarrhines, between 43.2 and 29.4 million years ago, and in the ancestor of lemuriformes, i.e. the strepsirrhine clade that inhabit Madagascar, between 59.3 and 55 million years ago. We also found species-specific retropseudogenes in the Philippine tarsier (*Carlito syrichta*), Bolivian squirrel monkey (*Saimiri boliviensis*), capuchin monkey (*Cebus capucinus imitator*) and vervet (*Chlorocebus sabaeus*). Although annotated sequences are not putatively functional, the identification of a retropseudocopy overlapping a lncRNA opens a new avenue to investigate TARDBP gene regulation.

## Results

### Multiple retropseudogenes lineages characterize the evolution of the TARDBP gene in primates

**Figure 1:**
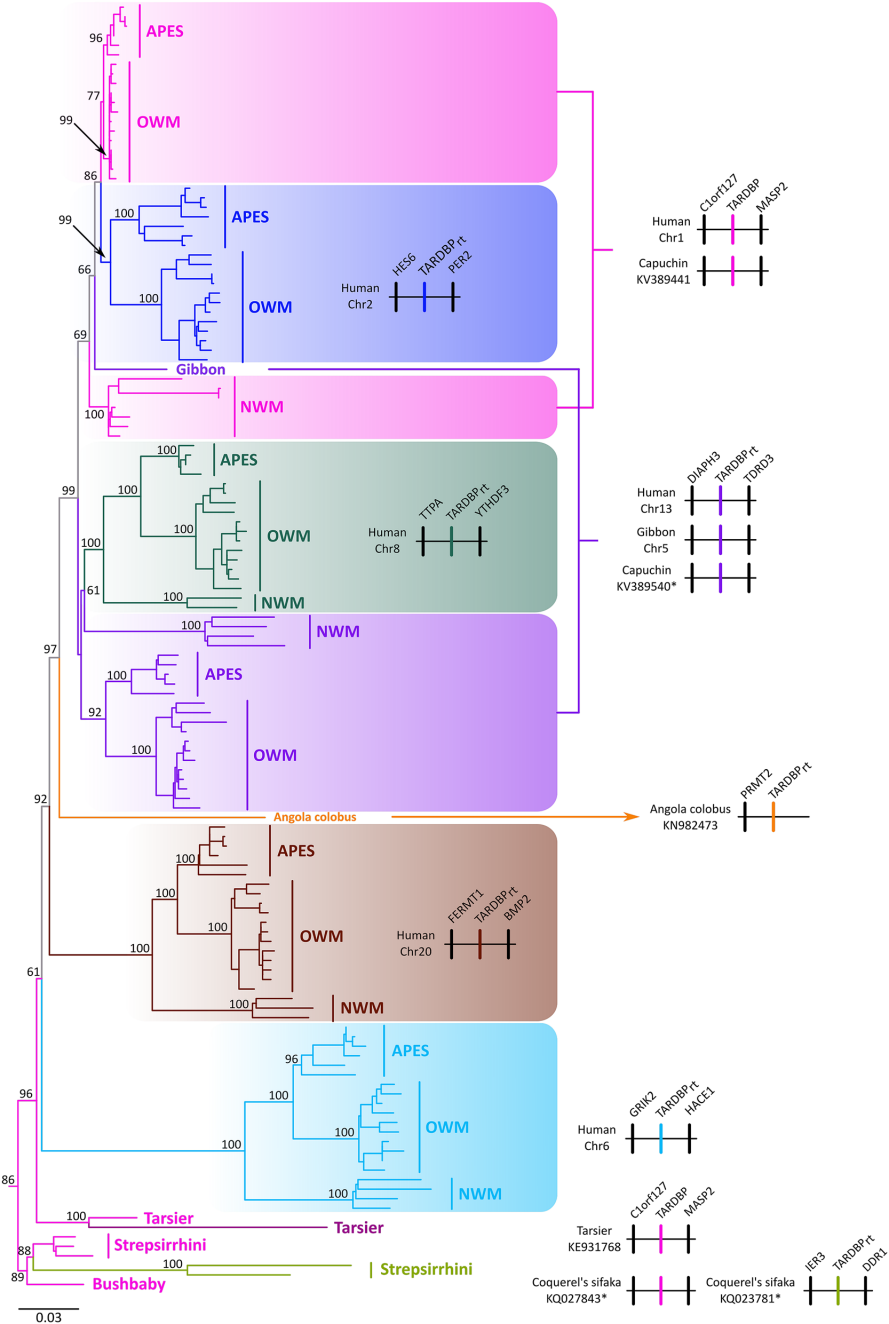
Maximum likelihood tree showing sister group relationships between the functional copy of TARDBP and primate retropseudogenes. Numbers on the nodes correspond to support values, i.e. the confidence of each node. TARDBP sequences from the African elephant (*Loxodonta africana*), blue whale (*Balaenoptera musculus*) and red fox (*Vulpes vulpes*) were used as outgroups (not shown). The scale denotes substitutions per site and colors represent lineages. The pink lineage represents the TARDBP functional copy. Synteny information is provided for each lineage at the right side of the figure.

According to our phylogenetic and synteny analyses, we identified retropseudogenes of the TARDBP gene that originated at different times during the evolution of primates. We identified retropseudogenes originated in the ancestor of anthropoids, between 67 and 43.2 million years ago, in the ancestor of catarrhines, between 43.2 and 29.4 million years ago, and in the ancestor of lemuriformes, i.e. the strepsirrhine clade that inhabit Madagascar, between 59.3 and 55 million years ago (Fig. 1). More recently in evolutionary time, we found species-specific retropseudogenes in the Philippine tarsier (*Carlito syrichta*), Bolivian squirrel monkey (*Saimiri boliviensis*), capuchin monkey (*Cebus capucinus imitator*) and vervet (*Chlorocebus sabaeus*). All of them did not have intron sequences and were identified on a different autosome in comparison to the chromosomal location of the functional copy (Fig. 1). Our gene tree did not significantly deviate from the most updated phylogenetic hypotheses for the main group of primates^31–33^, suggesting that the functional copy of the TARDBP gene was present as a simple copy gene in the ancestor of the group (Fig. 1).

We recovered three highly supported monophyletic groups containing representative species of all major groups of anthropoids i.e. apes, Old World monkeys and New World monkeys (light blue, brown and green lineages, Fig. 1), indicating that these retropseudogenes originated in the ancestor of the group, between 67 and 43.2 millions of years ago, and were maintained in representative species of all descendant primate groups. The retropseudogene lineage depicted with the purple shading (Fig. 1), although it was not recovered monophyletic, our synteny analyses suggest that it indeed belongs to a single lineage (Fig. 1). Representative species of the three purple clades possess the same flanking genes, DIAPH3 at the 5´ side and TDRD3 at the 3´ side of the retropseudogene, strongly suggesting that the lack of monophyly could be attributed to a phylogenetic artifact (Fig. 1). The small number of changes, as illustrated by the short branches that define the sister group relationships of the main clades, could be the main cause (Fig. 1). We also found a retropseudogene lineage that according to our phylogenetic tree originated in the ancestor of catarrhine primates, the group that includes apes and Old World monkeys (blue lineage, Fig. 1), between 43.2 and 29.4 million years ago. In this case, we recovered a clade containing the functional copy of the TARDBP gene in catarrhines (upper pink lineage, Fig. 1), sister to a group containing a retropseudogene in the same primate group (blue lineage, Fig. 1). The clade containing TARDBP functional sequences from New World monkeys was recovered sister to the above mentioned clade (Fig. 1). In this clade in addition to the functional TARDBP copy, we found New World monkey specific retropseudogenes for which the evolutionary history is difficult to resolve given the shortness of the branches (Fig. 1). We identified three retropseudogenes, two in the capuchin monkey (*Cebus capucinus imitator*) and one in the Bolivian squirrel monkey (*Saimiri boliviensis*). Finally, we recovered a sequence from the Angola colobus (*Colobus angolensis*)(yellow branch, Fig. 1), which was recovered sister to a clade containing the TARDBP functional copy (pink lineage, Fig. 1) and three retropseudogenes lineages (blue, purple and green clades, Fig. 1). The phylogenetic position of this branch in our gene tree suggests that it represents a retropseudogene originated in the anthropoid ancestor, but only conserved in this species. In support of this claim, the single flanking gene (PRMT2) found in the genomic piece containing the TARDBP retropseudogene in the Angola colobus is not shared with any other gene lineage described in this study (Fig. 1). In all cases, the identified retropseudogenes during the evolutionary history of anthropoid primates have premature stop codons, insertions and/or deletions (supplementary Figs. 1–5).

We also identified retropseudogenes in tarsiers and strepsirrhines (Fig. 1). We found a single retropseudogene in the Philippine tarsier (*Carlito syrichta*), which shows the hallmark of a sequence free from selective constraints, i.e., a long branch as a signal of an accelerated rate of evolution in comparison to the functional copy (Fig. 1). In the strepsirrhine clade we identified a highly supported lineage containing the TARDBP functional copy in three species, greater bamboo lemur (*Prolemur simus*), coquerel's sifaka (*Propithecus coquereli*) and the mouse lemur (*Microcebus murinus*), which in turn was recovered sister to an also highly supported clade containing retropseudogenes in the greater bamboo lemur (*Prolemur simus*) and coquerel's sifaka (*Propithecus coquereli*) (Fig. 1). This tree topology suggests that this retrocopy originated in the ancestor of lemuriformes, i.e. the strepsirrhine clade that inhabits Madagascar, between 59.3 and 55 million years ago, and it has been maintained in the genome of descendant species. Finally, the functional copy of the bushbaby (*Otolemur garnettii*) was recovered sister to the lemuriformes clade. Similar to the case of anthropoids, all retropseudogenes identified in tarsiers and strepsirrhines have premature stop codons, insertions and/or deletions (supplementary Figs. 6 and 7).

**Figure 2:**
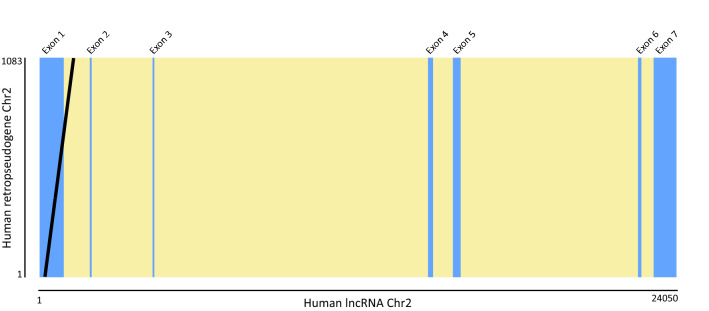
Graphical representation of the alignment between the lncRNA (ENSG00000225057)(x-axis) and the retropseudogene (y-axis) sequences of humans located on chromosome 2. Light blue and light yellow vertical rectangles denote exons and introns, respectively. The black diagonal line indicates a locally alignable region of high sequence identity.

Regarding the location of the retropseudocopies, most of them are within intergenic regions (Table 1). The exception is the one located on chromosome 2, which overlaps with a lncRNA gene (Fig. 2). Specifically, the retropseudocopy starts on position 198 of the first lncRNA exon and finishes on position 375 of the first lncRNA intron.

**Table 1:** Information regarding the genomic location of the TARDBP functional copy and retropseudocopies in humans.

Chromosome	Type	Genomic coordinates	Region type	Flanking genes	Orientation
1	Functional copy	11012344–11030528	Intergenic	C1orf127-TARDBP-MASP2	Forward
2	Retropseudocopy	238231881–238232964	lncRNA*	ILKAP-RPC-HES6	Forward
6	Retropseudocopy	102453,143–102454069	Intergenic	GRIK2-RPC-HACE1	Reverse
8	Retropseudocopy	63136407–63138084	Intergenic	TTPA-RPC-THDF3	Reverse
13	Retropseudocopy	6274779–60276011	Intergenic	DIAPH3-RPC-TDRD3	Forward
20	Retropseudocopy	6200989–6202191	Intergenic	FERMT1-RPC-BMP2	Reverse

*The ID of the lncRNA is ENSG00000225057; RPC, retropseudocopy.

## Discussion

**Figure 3:**
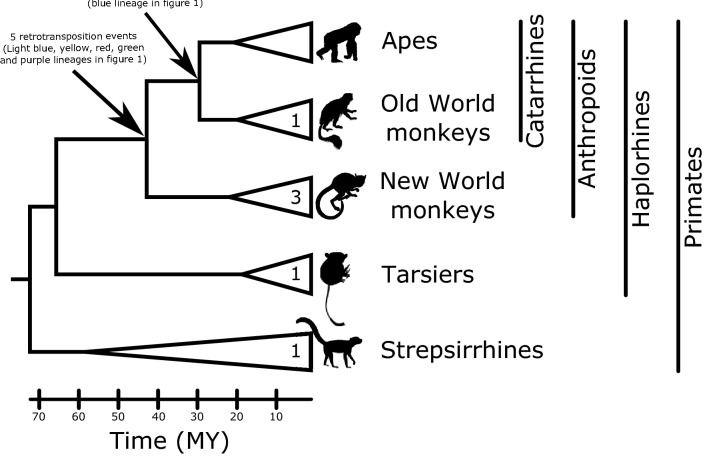
Time calibrated primate phylogeny showing the origin of the different retropseudogene lineages depicted in Fig. 1. Triangles represent the diversification of each primate group, where the left side angle defines the ancestor of each group. Numbers on the triangles correspond to retropseudogenes originated within a particular group of primates. Silhouette images were obtained from PhyloPic (http://phylopic.org/). Divergence times were obtained from timetree (http://www.timetree.org/).^37^.

In this study we revealed that the evolutionary history of TARDBP, a gene that in humans encodes TDP-43, an RNA-binding protein involved in several neurodegenerative disorders^13,14^, is characterized by the presence of retropseudogenes that originated at different ages during the evolutionary history of primates. An important fraction of the retropseudogenes originated in the anthropoid ancestor, between 67 and 43.2 million years ago, and has remained in the genome of the species (Fig. 3). This phenomenon fits the expectation of a peak of retrocopy formation around 40 million years ago, which coincides with an increased activity of L1 retroelements that produced an increment in SINE/Alu retrocopy repeat amplification^29,30^. Interestingly, this period of time represents a key moment during the evolutionary history of primates, the radiation of the anthropoid lineage, where significant morphological and physiological traits arose^34^. Thus, this period of Vesuvian mode of evolution could be seen as a source of evolutionary novelty that fueled the origin of the phenotypes that define the anthropoid lineage^2,35,36^. Other retropseudogenes originated in the catarrhine ancestor, between 43.2 and 29.4 million years ago, and in other primate groups (Fig. 3).

In agreement with the literature, and given the nature of the process originating retrocopies, all of them seem to be non-functional as canonical TARDBP^3^, which can be verified by the presence of insertions, deletions and/or premature stop codons (supplementary Figs. 1 and 7). The identification of several retropseudogenes for the TARDBP gene in primates appears to be not a surprise as this gene complies with all the requisites to be a gene with multiple retropseudogenes^38–40^, i.e., short transcripts (coding for 61 to 414 amino acids)^41^, widely and highly expressed^42^, low GC-content (47%, average among 23 primate species) and highly conserved (3.4%, maximal divergence among primates). Furthermore, in agreement with the slow rate of pseudogene length shortening over time, the identified retropseudogenes possess a length (mean 1128 bp, median 1193 bp) similar to the functional TARDBP gene (1245 bp).

Among apes, the number of TARDBP retrotransposition events appear to be higher in comparison to the average number of retrocopies per parental gene in their genomes^43^. On average, ape genomes possess 2.9 retrocopies per parental gene^43^, however in our study we identified five TARDBP retropseudogenes in each examined ape species. Coincident with previous evidence, we also found a higher number of retropseudogenes in New World monkeys^43^. Although it is not clear why this group of primates has more retrocopies compared to catarrhines, it is suggested that a specific lineage expansion of L1PA1 and L1P3 subelements could be related to the observed pattern^2,43^.

TDP-43 binds to long clusters of GU-rich RNA sequences, which in humans are found in one-third of transcribed genes^44^. This allows TDP-43 to regulate the processing of thousands of transcripts, including that of its own transcript^45^. In fact, TDP-43 establishes a tightly regulated feedback loop^46^. It has been demonstrated that a twofold increase or decrease in TDP-43 levels is sufficient to promote neurodegeneration^45^. Thus, TARDBP retropseudogenes could represent an additional layer of regulation of TDP-43 levels and activity. In this regard, it seems interesting that one of the retropseudocopies is located in a lncRNA (Fig. 2), a pattern that appears not unusual in the human genome^47^. This fact opens the possibility that this retropseudocopy regulates the expression of the functional copy of TARDBP^48–50^. In fact, blast searches against the expressed sequence tags (est) database, which represent a snapshot of genes expressed in a given tissue and/or at a specific developmental stage, show at least one record (BI825397), which possesses an identity value of 90.5% with the retropseudocopy located on chromosome 2 and is expressed in medulla in an adult male. In contrast, with the TARDBP functional copy, the identity value is 70.6%. It will be important to determine whether in humans the levels of TDP-43 are affected by the levels of this lncRNA, in particular in the brain of patients suffering the aforementioned neurodegenerative disorders.

In conclusion, in this work, we demonstrate that the TARDBP gene in primates has an evolutionary history characterized by the presence of multiple retropseudogene lineages. In the ancestor of anthropoids occurred a significant increment of retrotransposition activity, which led to intronless sequences that cannot give rise to functional proteins. However, the fact that one of the retropseudocopies is present in a lncRNA and is transcribed opens the opportunity to investigate further its role in regulating the expression of the functional TARDBP gene copy, and its influence in the outcome or fate of the associated neurodegenerative disorders.

## Methods

### DNA sequences

#### DNA sequences and phylogenetic analyses

We performed searches for TAR DNA Binding Protein (TARDBP) genes in primate genomes in Ensembl v.102^41^. We retrieved primate orthologs, using the human (*Homo sapiens*) entry, based on the ortholog prediction function of Ensembl v.102^41^. We identified TARDBP retropseudogenes in primate species by performing BLASTN searches^51^, against the whole genome sequence in Ensembl v.102^41^ using default settings. In each case the query sequence (TARDBP) was from the same species of the genome in which retropseudogenes were looking for. In our searches, a retropseudogene was recognized as a sequence containing all exons together and found in a different chromosome in comparison to the functional copy. Genomic fragments containing retropseudogenes were extracted and manually annotated by comparing the coding sequence of the same species using the program Blast2seq v2.5^52^ with default parameters. Accession numbers and details about the taxonomic sampling are available in Supplementary Table S1.

Nucleotide sequences were aligned using MAFFT v.7^53^, allowing the program to choose the alignment strategy (FFT-NS-i). We used the proposed model tool of IQ-Tree v.1.6.12^54^ to select the best-fitting model of nucleotide substitution, which selected GTR + F + R3. We used the maximum likelihood method to obtain the best tree using the program IQ-Tree v1.6.12^55^. We assessed support for the nodes using three strategies: a Bayesian-like transformation of aLRT (aBayes test)^56^, SH-like approximate likelihood ratio test (SH-aLRT)^57^ and the ultrafast bootstrap approximation^58^. We carried out 20 independent runs to explore the tree space, and the tree with the highest likelihood score was chosen. TARDBP sequences from the African elephant (*Loxodonta africana*), blue whale (*Balaenoptera musculus*) and red fox (*Vulpes vulpes*) were used as outgroups.

#### Assessment of conserved synteny

We examined genes found upstream and downstream of functional copies and retropseudogenes. We used the estimates of orthology and paralogy derived from the Ensembl Compara database^59^; these estimates are obtained from a pipeline that considers both synteny and phylogeny to generate orthology mappings. These predictions were visualized using the program Genomicus v100.01^60^. Our assessments were performed in representative species for each lineage.

## Supplementary Information

Supplementary Information 1.

Supplementary Figure 1.

Supplementary Figure 2.

Supplementary Figure 3.

Supplementary Figure 4.

Supplementary Figure 5.

Supplementary Figure 6.

Supplementary Figure 7.

Supplementary Table 1.

Supplementary Information 2.
