# Supplementary material for: Identification of multiple TAR DNA binding protein retropseudogene lineages during the evolution of primates
Source: Sci Rep. 2022 Mar 9;12:3823. doi: 10.1038/s41598-022-07908-8 (PMC8907276; doi:10.1038/s41598-022-07908-8)
Supplement: Supplementary file 2 — Supplementary Figure 1. [file 41598_2022_7908_MOESM2_ESM.pdf]

```
                  1        10          20          30          40          50          60          70         80          90
Human Chr1 TARDBP ATG TCT GAA TAT ATT CGG GTA ACC GAA GAT GAG AAC GAT GAG CCC ATT GAA ATA CCA TCG GAA GAC GAT -GG GAC GGT GCT GCT CTC CAC
                   M   S   E   Y   I   R   V   T   E   D   E   N   D   E   P   I   E   I   P   S   E   D   D  - G   T   V   L   L   S   T
Human Chr2        ATG TCT GAA TAT ATT CGG GTA ACC GAA GAT GAG AAC GAT GAG CCC ATT GAA ATA CCA TCG GAA GAC GAT -GG GAC GGT GCT GCT GTC CAC
                   M   S   E   Y   I   R   V   T   E   D   E   N   D   E   P   I   E   I   P   S   E   D   D  - G   T   V   L   L   S   T
Macaque Chr12     GTG TCT GAA TAT ATT CAG GTA ACC GAA GAT GAG AAC GAT GAG CCC ATT GAA ATA TCA TCG CAA GAC GAT GGG GAC AGT GCT GCT GTC CAC
                   V   S   E   Y   I   Q   V   T   E   D   E   N   D   E   P   I   E   I   S   S   Q   D   D   G   D   S   A   A   V   H

                              100         110         120         130         140         150         160         170         180
Human Chr1 TARDBP GGT TAC AGC CCA GTT TCC AGG GGC GTG TGG GCT TCG CTA CAG GAA TCC AGT GTC TCA GTG TAT GAG AGG TGT CCG GCT GGT AGA AGG AAT
                    V   T   A   Q   F   P   G   A   C   G   L   R   Y   R   N   P   V   S   Q   C   M   R   G   V   R   L   V   E   G   I
Human Chr2        GGT TAC AGC CCA GTT TCC AGG GGC GTG TGG GCT TCG CTG CAG GAA TCC GGT GTC TCA ATG TAT GAG AGG TGT CCG GCT GGT AGA AGG AAT
                    V   T   A   Q   F   P   G   A   C   G   L   R   C   R   N   P   V   S   Q   C   M   R   G   V   R   L   V   E   G   I
Macaque Chr12     GCT TAT GGC ACA GTT TCC AGG GGC GTG TGG GCT TCG CTA CAG AAA TCC AGC GTC TCA ATG TAT GAG AGG TGT ACG GCT GGT AGA AGG AAT
                   A   Y   G   T   V   S   R   G   V   W   A   S   L   Q   K   S   S   V   S   M   Y   E   R   C   T   A   G   R   R   N

                              190         200         210         220         230         240         250         260         270
Human Chr1 TARDBP TCT GCA TGC CCC AGA TGC TGG CTG GGG AAA TCT GGT GTA TGT TGT CAA CTA TCC AAA AGA TAA CAA AAG AAA AAT GGA TGA GAC AGA TGC
                    L   H   A   P   D   A   G   W   G   N   L   V   Y   V   V   N   Y   P   K   D   N   K   R   K   M   D   E   T   D   A
Human Chr2        TCT GCA TGC CCC CGA TGC TGG CTG GAG AAA TCC GGT GTA TGT TGT CAA CTA TCC GAA AGA TAA CAA AAG AAA AAT GGG TGA GAC AGA AGC
                    L   H   A   P   D   A   G   W   R   N   P   V   Y   V   V   N   Y   P   K   D   N   K   R   K   M   G   E   T   E   A
Macaque Chr12     TCT GCC TGC CCC CGA TGC TGG CTG GGG AAA TCT GGT GTA TGT TGT CAA CTA TCC GAA AGA TAA CAA AAG AAA AAT GGA TGA GAC AGA TGC
                   S   A   C   P   R   C   W   L   G   K   S   G   V   C   C   Q   L   S   E   R   *   Q   K   K   N   G   *   D   R   C

                              280         290         300         310         320         330         340         350         360
Human Chr1 TARDBP TTC ATC AGC AGT GAA AGT GAA AAG AGC AGT CCA GAA AAC ATC CGA TTT AAT AGT GTT GGG TCT CCC ATG GAA AAC AAC CGA ACA GGA CCT
                    S   S   A   V   K   V   K   R   A   V   Q   K   T   S   D   L   I   V   L   G   L   P   W   K   T   T   E   Q   D
Human Chr2        TTC ATC AGC AGT GAA AGT GAA AAG ACC AG- --- --- --- -TC CGA TTT AAT AGT GTT GGG TCT CCC ATG GAA AAC AAC TGA ACA GGA CCT
                    S   S   A   V   K   V   K   R   P  - --- --- --- -V   R   F   N   S   V   G   S   P   M   E   N   N   *   T   G   P
Macaque Chr12     TTC ATG AGC AGT GAA AGT GAA AAG AGT AGT CTA GAA AAC ATC CGA TTT AAC AGT GTT GGG TCT CCC GTG GGG AAC GAC TGA ACA GGG TCT
                   F   M   S   S   E   S   E   K   S   S   L   E   N   I   R   F   N   S   V   G   S   P   V   G   N   D   *   T   G   S
```

Supplementary figure 1. Nucleotide alignment of the TARDBP functional copy of humans (*Homo sapiens*) and TARDBP retrocopies in representative species of primates in which the retrocopy was identified corresponding to the blue lineage on figure 1. The shading highlights the mutations that make the retrocopies non-functional.
