# Supplementary material for: Identification of multiple TAR DNA binding protein retropseudogene lineages during the evolution of primates
Source: Sci Rep. 2022 Mar 9;12:3823. doi: 10.1038/s41598-022-07908-8 (PMC8907276; doi:10.1038/s41598-022-07908-8)
Supplement: Supplementary file 3 — Supplementary Figure 2. [file 41598_2022_7908_MOESM3_ESM.pdf]

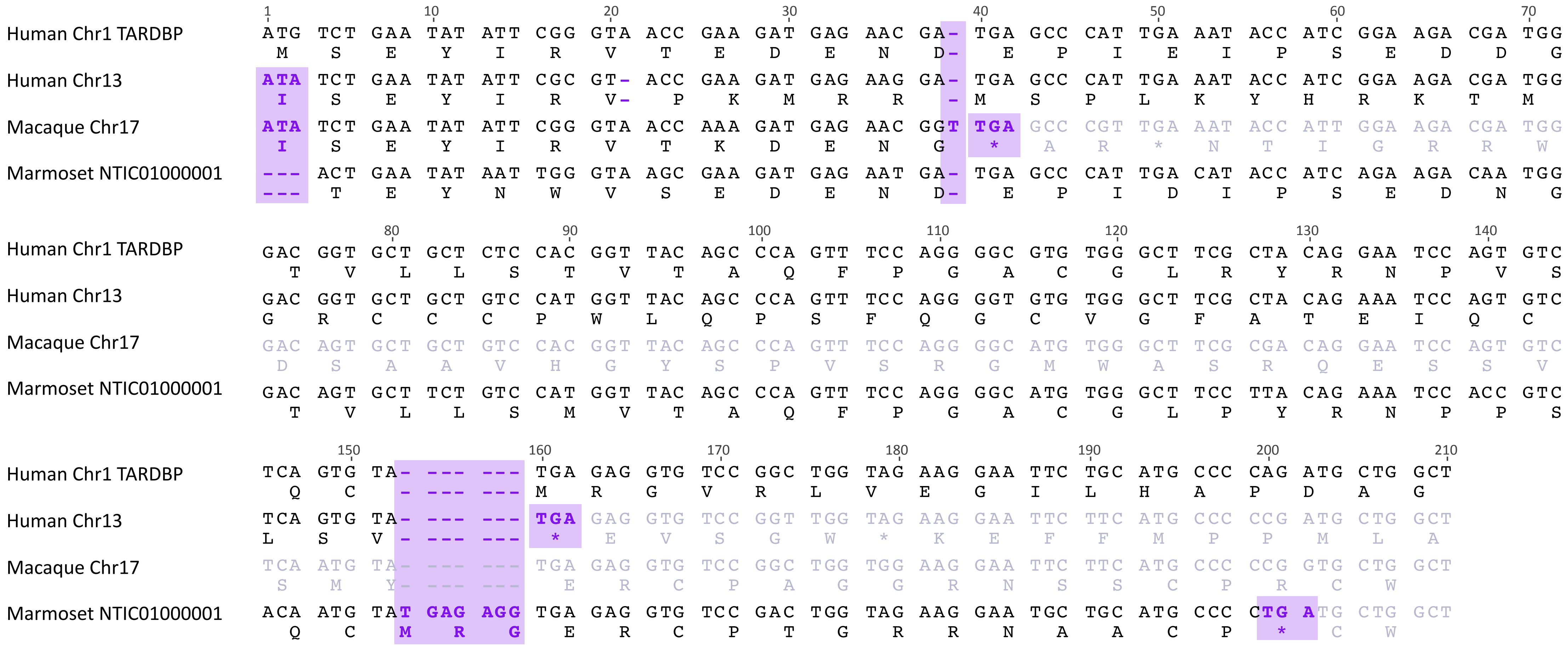

Supplementary figure 2. Nucleotide alignment of the TARDBP functional copy of humans (*Homo sapiens*) and TARDBP retrocopies in representative species of primates in which the retrocopy was identified corresponding to the purple lineage on figure 1. The shading highlights the mutations that make the retrocopies non-functional.
