# Supplementary material for: Identification of multiple TAR DNA binding protein retropseudogene lineages during the evolution of primates
Source: Sci Rep. 2022 Mar 9;12:3823. doi: 10.1038/s41598-022-07908-8 (PMC8907276; doi:10.1038/s41598-022-07908-8)
Supplement: Supplementary file 4 — Supplementary Figure 3. [file 41598_2022_7908_MOESM4_ESM.pdf]

```
                          1        10       20       30       40       50       60
Human Chr1 TARDBP         ATG TCT GAA TAT ATT CGG GTA ACC GAA GAT GAG AAC GAT GAG CCC ATT GAA ATA CCA TCG GAA
                           M   S   E   Y   I   R   V   T   E   D   E   N   D   E   P   I   E   I   P   S   E
Human Chr8                ATG TCT GAA TAT ATT CGG GTA ACC AAA GAT GAG AAC AAT GAG CCC ATT TAA ATA CCT TCA GAG
                           M   S   E   Y   I   R   V   T   K   D   E   N   N   E   P   I   *   I   P   S   E
Macaque Chr8              ATG TCT GAA TAG ATT CAG GTA ACC GAA GAT GAG AAC AAT TAG CCC ATT TAA ATA CCA CCA GAA
                           M   S   E   *   I   Q   V   T   E   D   E   N   N   *   P   I   *   I   P   P   E
Ma's night monkey KZ195588 ATA TTT GAA TAT ACT C-G GTA ACC AAA GAT GAG AAC AAT GAG CCC A-T GAA ATA CCA TTG GAA
                           I   F   E   Y   T  -R   *   P   K   M   R   T   M   S   P   -  *   N   T   I   G

                                  70       80       90        100   105
Human Chr1 TARDBP         GAC GAT GGG ACG GTG CTG CTC TCC ACG GTT ACA GCC CAG TTT
                           D   D   G   T   V   L   L   S   T   V   T   A   Q   F
Human Chr8                GAC GAA GGG ACG GTG CTG CTG TCC ACT GTT ACA GC- --- ---
                           D   E   G   T   V   L   L   S   T   V   T   -  --- ---
Macaque Chr8              AAC AAT GGG ACG GCG CTG CTG TCC ATG ATT ACG GC- --- ---
                           N   N   G   T   A   L   L   S   M   I   T   -  --- ---
Ma's night monkey KZ195588 GAT GAT GGG ACG GTG CTG CTG TCC ACA GTT ACA GC- --- ---
                           R   *   W   D   G   A   A   V   H   S   Y   S - --- ---
```

Supplementary figure 3. Nucleotide alignment of the TARDBP functional copy of humans (*Homo sapiens*) and TARDBP retrocopies in representative species of primates in which the retrocopy was identified corresponding to the green lineage on figure 1. The shading highlights the mutations that make the retrocopies non-functional.
