# Supplementary material for: Identification of multiple TAR DNA binding protein retropseudogene lineages during the evolution of primates
Source: Sci Rep. 2022 Mar 9;12:3823. doi: 10.1038/s41598-022-07908-8 (PMC8907276; doi:10.1038/s41598-022-07908-8)
Supplement: Supplementary file 5 — Supplementary Figure 4. [file 41598_2022_7908_MOESM5_ESM.pdf]

```
                            1           10          20          30          40          50          60          70          80          90
Human Chr1 TARDBP           ATG TCT GAA TAT ATT CGG GTA ACC GAA GAT GAG AAC GAT GAG CCC ATT GAA ATA CCA TCG GAA GAC GAT GGG ACG GTG CTG CTC TCC ACG
                             M   S   E   Y   I   R   V   T   E   D   E   N   D   E   P   I   E   I   P   S   E   D   D   G   T   V   L   L   S   T
Human Chr20                 ATG TCT GAA TAC ACT AGG GTA AGT GAA GTT GAG AAT AAG GAG CTC GTT GAA ATA TCA TCA GAA GAC AAT GGG GTG GTG CTG CTG TCC ACA
                             M   S   E   Y   T   R   V   S   E   V   E   N   K   E   L   V   E   I   S   S   E   D   N   G   V   V   L   L   S   T
Macaque Chr10               ATG TTT GAA TAC ACT TGG GTA AGT GAA GAT GAG AAT AAG GAG CTC ATG GAA ATA TCA TCA GAA TAC AAT GGG GTA GTG CTG CCG TCC ACA
                             M   F   E   Y   T   W   V   S   E   D   E   N   K   E   L   M   E   I   S   S   E   Y   N   G   V   V   L   P   S   T
Marmoset NTIC01034389       ATG TCT GAA TAT ACT CAA GTA ACT GAA GAT GAG AAT AAG GAA CTC TTT GAA AT- --A TCA GAA GAC AAT GGG ATG GTG CTG CTG TCC ATG
                             M   S   E   Y   T   Q   V   T   E   D   E   N   K   E   L   F   E   I-  --  S   E   D   N   G   M   V   L   L   S   M

                                        100         110         120         130         140         150         160         170         180
Human Chr1 TARDBP           GTT ACA GCC CAG TTT CCA GGG GCG TGT GGG CTT CGC TAC AGG AAT CCA GTG TCT CAG TGT ATG AGA GGT GTC CGG CTG GTA GAA GGA ATT
                             V   T   A   Q   F   P   G   A   C   G   L   R   Y   R   N   P   V   S   Q   C   M   R   G   V   R   L   V   E   G   I
Human Chr20                 GTT ACA GCC CAG TTT CCA GGG GCA TGT GGA CTT CAC TAC AAG AAT CCA GTG TCT CAG TGT ATG AGA GGT ATC CAG CTG GTA GAA GGA ATT
                             V   T   A   Q   F   P   G   A   C   G   L   H   Y   K   N   P   V   S   Q   C   M   R   G   I   Q   L   V   E   G   I
Macaque Chr10               GTT ACA GCC CAG TTT CCA GGG GCA CGT GAG CTC CAC CAC AAG AAT GCA GTG TCT CAA TGT ATG AAA GGT ATC CTG CTG GTA GAA GGA ATT
                             V   T   A   Q   F   P   G   A   R   E   L   H   H   K   N   A   V   S   Q   C   M   K   G   I   L   L   V   E   G   I
Marmoset NTIC01034389       GGT ACA GTC CAG TTT CCA GGG GCA TGT GGG CTT CAC TAC AAG AAT CCA GTG TCT CAA TGT ATG AGA GGT ATC TAG TTG GCA GAA GGA ATT
                             G   T   V   Q   F   P   G   A   C   G   L   H   Y   K   N   P   V   S   Q   C   M   R   G   I   *   L   A   E   G   I

                                        190         200         210         220         230         240         250         260         270
Human Chr1 TARDBP           CTG CAT GCC CCA GAT GCT GGC TGG GGA AAT CTG GTG TAT GTT GTC AAC TAT CCA AAA GAT AAC AAA AGA AAA ATG GAT GAG ACA GAT GCT
                             L   H   A   P   D   A   G   W   G   N   L   V   Y   V   V   N   Y   P   K   D   N   K   R   K   M   D   E   T   D   A
Human Chr20                 CTG CAT G-C CCT GAT GCT GAC TGT GGA AAT CTG GTA TAT GTT GTC AAC TGT CCC AAA GAT AAC AAA AGA AAA ATG GGT GAG A-- --- ---
                             L   H  -A   L   M   L   T   V   E   I   W   Y   M   L   S   T   V   P   K   I   T   K   E   K   W   V   R   --  --- ---
Macaque Chr10               CTG CAT GCC CCT GAT GCT GAC AGT GGA AAT CTG GTA TAT GTT GTC AAC TGT CCC AAA GAC AAC AAA AGA AAA ATG GGT GAG A-- --- ---
                             L   H   A   P   D   A   D   S   G   N   L   V   Y   V   V   N   C   P   K   D   N   K   R   K   M   G   E   --  --- ---
Marmoset NTIC01034389       CTG CAT GCC CCT CAT GCT GCC TGT GGA AAT CTG GTC TAT GTT GTC AAC TAT CCC AAA GCC AAC AAA AGA AAA ATG GAT GAG A-- --- ---
                             L   H   A   P   H   A   A   C   G   N   L   V   Y   V   V   N   Y   P   K   A   N   K   R   K   M   D   E   --  --- ---

                                        280         290         300         310         320         330         340         350
Human Chr1 TARDBP           TCA TCA GCA GTG AAA GTG AAA AGA GCA GTC CAG AAA ACA TCC GAT TTA ATA GTG TTG G-- --- --- --- --- --- --- --
                             S   S   A   V   K   V   K   R   A   V   Q   K   T   S   D   L   I   V   L   --  --- --- --- --- --- --- --
Human Chr20                 --- -CG GCA GTG AAA GTG --A AGA GCA GTC CAG AAA ATA TCT TAT TTA ATA GCG TTG GTA ATT AGT GAG TTT TTG CTC TC
                            --- -   R   Q   *   K   *   --  R   A   V   Q   K   I   S   Y   L   I   A   L   V   I   S   E   F   L   L
Macaque Chr10               --- -CA GCA GTG AAA GTG --A AGA GCA GTC CAG AAA ATA TCT TAC TTA ATA GTA CTG GTA ATT AGT GAG TTT GTG CTC TG
                            --- -T   A   V   K   V   --  K   S   S   P   E   N   I   L   L   N   S   T   G   N   *   *   V   C   A   L
Marmoset NTIC01034389       --- -CA TCA GTG AAA GTG --A AGA GCA GCC CAG AAA ACA TCT TAT TTA ATA GTG TTG GTA ATT AGC GAG TTT GTG --C TG
                            --- -T   S   V   K   V   --  K   S   S   P   E   N   I   L   F   N   S   V   G   N   *   R   V   C  --A
```

**Supplementary figure 4.** Nucleotide alignment of the TARDBP functional copy of humans (*Homo sapiens*) and TARDBP retrocopies in representative species of primates in which the retrocopy was identified corresponding to the brown lineage in figure 1. The shading highlights the mutations that make the retrocopies non-functional.
