# Supplementary material for: Identification of multiple TAR DNA binding protein retropseudogene lineages during the evolution of primates
Source: Sci Rep. 2022 Mar 9;12:3823. doi: 10.1038/s41598-022-07908-8 (PMC8907276; doi:10.1038/s41598-022-07908-8)
Supplement: Supplementary file 7 — Supplementary Figure 6. [file 41598_2022_7908_MOESM7_ESM.pdf]

```
                        1           10           20            30            40           50           60            70           80           90
Tarsier KE931768 TARDBP ATG TCT GAA TAT ATT CGG GTA ACT GAA GAC GAG AAT GAT GAG CCC ATT GAG ATA CCA TCA GAA GAT GAT GGG ACG GTA CTG CTG TCC ACG
                         M   S   E   Y   I   R   V   T   E   D   E   N   D   E   P   I   E   I   P   S   E   D   D   G   T   V   L   L   S   T
Tarsier KE944022        --- TCT GAA TAT ATT CGG GTA ACT GAA GAC --- --- --- --- --- --- -AA ATA CCA GCA GAA GAT GAT GGG GAG GTA CTA CTG TTC CTG
                        ---  S   E   Y   I   R   V   T   E   D  --- --- --- --- --- --- -  K   Y   Q   Q   K   M   M   G   R   Y   Y   C   S   W

                                    100          110          120           130          140          150           160          170          180
Tarsier KE931768 TARDBP GTC ACG GCC CAG TTT CCA GGA GCA TGT GGG CTG CGC TAC CGG AAT CCA GTG TCG CAG TGT ATG AGA GGT GTC CGA CTG GTG GAA GGA ATC
                         V   T   A   Q   F   P   G   A   C   G   L   R   Y   R   N   P   V   S   Q   C   M   R   G   V   R   L   V   E   G   I
Tarsier KE944022        GTC ACA GCC CAG TTT CCA GGG GCA TGT GGG CCG TGC TAC AGG AAT TCA GTG TCT CAA TGT ATG AGA GAT ATC TGA CTG GAA GAA GGA ATC
                          S   Q   P   S   F   Q   G   H   V   G   R   A   T   G   I   Q   C   L   N   V   *   E   I   S   D   W   K   K   E
```

Supplementary figure 6. Nucleotide alignment of the TARDBP functional copy of the philippine tarsier (*Carlito syrichta*) and TARDBP retrocopy identified in the same species. The shading highlights the mutations that make the retrocopy non-functional.
