# Supplementary material for: Identification of multiple TAR DNA binding protein retropseudogene lineages during the evolution of primates
Source: Sci Rep. 2022 Mar 9;12:3823. doi: 10.1038/s41598-022-07908-8 (PMC8907276; doi:10.1038/s41598-022-07908-8)
Supplement: Supplementary file 8 — Supplementary Figure 7. [file 41598_2022_7908_MOESM8_ESM.pdf]

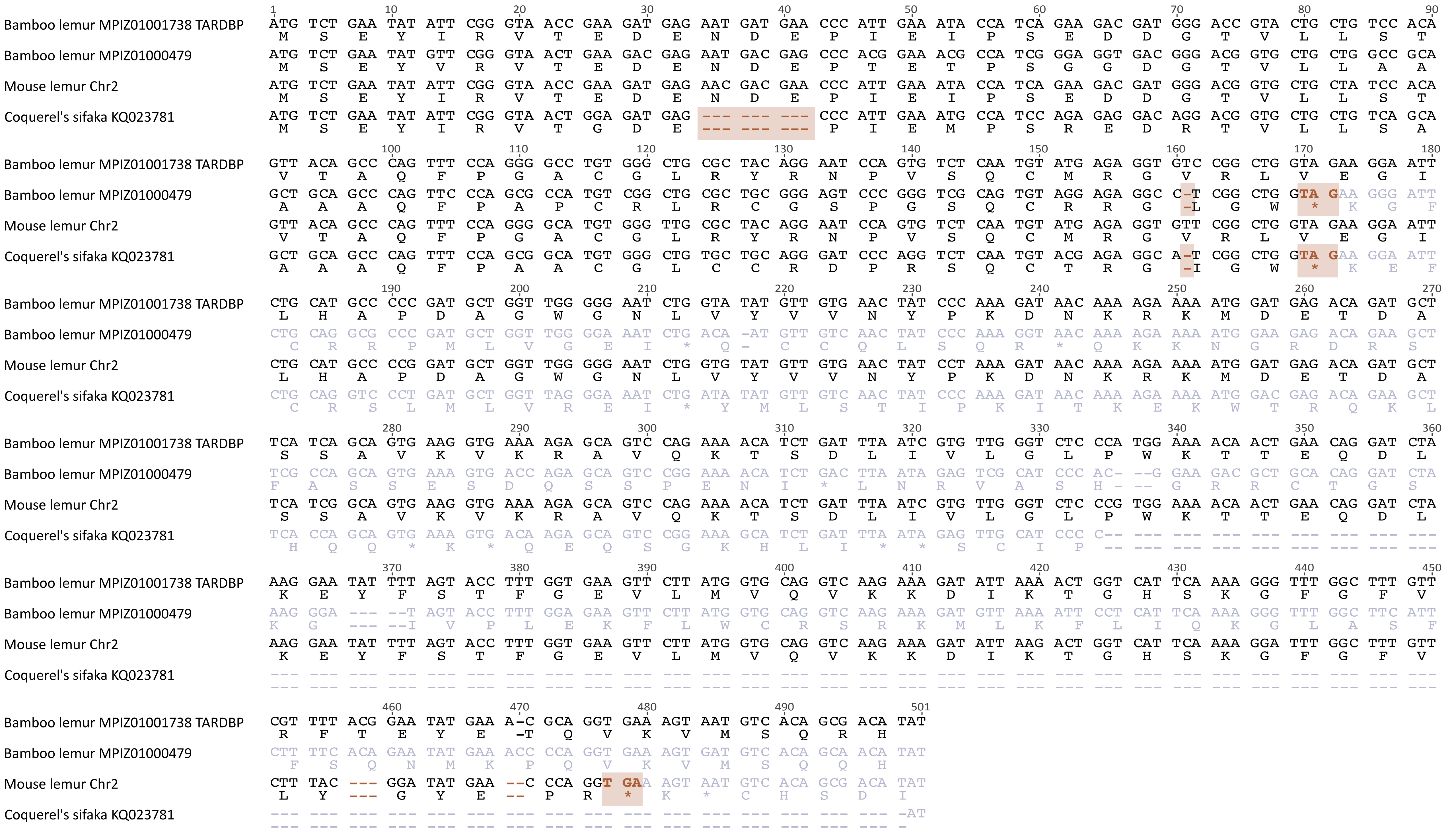

Supplementary figure 7. Nucleotide alignment of the TARDBP functional copy of the bamboo lemur (*Prolemur simus*) and TARDBP retrocopies in representative species of strepsirrhines in which the retrocopy was identified on figure 1. The shading highlights the mutations that make the retrocopies non-functional.
